# Development and Validation of a Prenatal Doppler Hemodynamic–Clinical Risk Model for Predicting Adverse Perinatal Outcomes in Fetuses with Umbilical Artery Thrombosis

**DOI:** 10.3390/jcm15176779

**Published:** 2026-09-01

**Authors:** Peng Tu, Cheng Chen, Xiaohang Zhang, Xuemei Zhang, Suzhen Ran

**Affiliations:** 1 Department of Ultrasound, Chongqing Health Center for Women and Children, Chongqing 401147, China; tp0126@hospital.cqmu.edu.cn (P.T.); cc@hospital.cqmu.edu.cn (C.C.); zxh5678@hospital.cqmu.edu.cn (X.Z.); swinters789@hospital.cqmu.edu.cn (X.Z.); 2 Department of Ultrasound, Women and Children’s Hospital of Chongqing Medical University, Chongqing 401147, China; 3 NHC Key Laboratory of Birth Defects and Reproductive Health, Chongqing 401147, China

**Keywords:** umbilical artery thrombosis, adverse outcomes, prediction model

## Abstract

**Background/Objectives:** Umbilical artery thrombosis (UAT) is a rare but potentially catastrophic obstetric complication associated with a high risk of adverse perinatal outcomes. However, clinically applicable risk stratification tools for pregnancies complicated by UAT remain limited. Therefore, this study aimed to develop and validate a multivariable clinical prediction model integrating maternal clinical characteristics, fetal characteristics, and Doppler-derived hemodynamic parameters for fetuses with UAT. The model may facilitate early risk stratification and support individualized prenatal management and clinical decision-making. **Methods:** This retrospective study included 114 fetuses with UAT at the Women and Children’s Hospital of Chongqing Medical University between January 2017 and January 2025. Candidate predictors were predefined based on previous literature, clinical knowledge, and their potential associations with adverse pregnancy outcomes in UAT, encompassing maternal characteristics, UAT-related factors, Doppler-derived hemodynamic parameters, and relevant clinical factors. Predictors were selected using LASSO with 10-fold cross-validation and entered into multivariable logistic regression to construct a nomogram. Model discrimination, calibration, and clinical utility were assessed using AUC, calibration plots, and decision curve analysis, respectively. The optimal cutoff was determined by the maximum Youden index. Bootstrap resampling was used for internal validation and generalizability assessment. **Results:** Of the 114 fetuses, 57 (50.0%) experienced adverse pregnancy outcomes. The final nomogram incorporated maternal age, umbilical artery pulsatility index (UA-PI), ductus venosus pulsatility index (DV-PIV), uterine artery pulsatility index (UtA-PI), gravidity, diabetes mellitus, fetal growth restriction (FGR), and UAT laterality. Higher UA-PI (OR, 7.15; 95% CI, 1.01–50.57), higher DV-PIV (OR, 13.18; 95% CI, 2.34–74.41), and FGR (OR, 7.53; 95% CI, 1.77–31.97) were associated with increased odds of adverse outcomes, while UAT laterality showed a borderline association (OR, 2.61; 95% CI, 1.00–6.81). The nomogram achieved an AUC of 0.791 (95% CI, 0.706–0.876), with 70.2% sensitivity and 78.9% specificity at the optimal cutoff. Bootstrap calibration showed a mean absolute error of 0.042. Decision curve analysis demonstrated a positive net benefit across threshold probabilities of 11–92%. **Conclusions:** This study establishes an internally validated model for predicting adverse perinatal outcomes in fetuses with UAT using clinical characteristics and gestational age-adjusted Doppler hemodynamic parameters available at the time of diagnosis. The model may provide a basis for risk stratification and individualized antenatal surveillance.

## 1. Introduction

Umbilical artery thrombosis (UAT) is a rare condition caused by thrombotic occlusion of one umbilical artery (UA), often leading to serious perinatal complications such as intrauterine hypoxia and fetal demise [1,2]. Although several studies [3] have proposed predictive models for adverse outcomes in UAT, most have focused on maternal factors (gestational diabetes mellitus (GDM), coagulopathies, etc.) and fetal conditions (umbilical cord (UC) anomalies, placental inflammation, etc.), without incorporating real-time fetal hemodynamic assessments. Furthermore, these models often suffered from limited sample sizes, which substantially reduces their clinical applicability.

In our retrospective study [4], we identified a strong association between ultrasonographic hemodynamic parameters and adverse pregnancy outcomes, highlighting the value of hemodynamic monitoring for early detection of UAT. Absent or reversed end-diastolic flow in the UA typically reflects sustained vasoconstriction within the placental villous vasculature, indicating advanced placental insufficiency [5,6]. This hemodynamic disturbance is closely linked to fetal growth restriction (FGR) and other adverse perinatal outcomes [7,8]. In response to hypoxia secondary to placental dysfunction, the middle cerebral artery (MCA) may undergo compensatory vasodilation—a phenomenon known as the ‘brain-sparing effect’—to maintain perfusion to vital organs [9,10]. Additionally, a decreased or absent a-wave in the ductus venosus (DV) is a recognized early marker of severe hypoxemia and metabolic acidosis, often reflecting impaired myocardial function and increased cardiac afterload [11,12].

Given the paucity of data on fetuses with UAT, determining the optimal timing of delivery remains a significant clinical challenge [13,14]. This study aims to enhance predictive accuracy by integrating maternal clinical parameters, fetal baseline characteristics, and Doppler-derived hemodynamic indices. A multi-parametric predictive model was developed to precisely assess the risk of adverse outcomes in fetuses with UAT, thereby supporting personalized clinical decision-making and improving perinatal outcomes.

## 2. Methods

### 2.1. Study Participants

This historical cohort study included fetuses diagnosed with UAT at the Women and Children’s Hospital of Chongqing Medical University between January 2017 and January 2025.

This study received two approvals from the Ethics Committee of Women and Children’s Hospital of Chongqing Medical University/Chongqing Health Center for Women and Children: the initial approval was granted on 16 October 2023 (Approval Code: 2023 (013)), and the subsequent approval was granted on 23 July 2025 (Approval Code: 2025 (73)), and written informed consent was obtained from all participants. Fetuses were followed until delivery or pregnancy termination.

### 2.2. Information Collection

#### 2.2.1. Basic Clinical Information

A structured case questionnaire was designed to collect baseline clinical data from pregnant women, including demographic information (maternal age, gestational weight gain, body mass index, etc.), medical history (GDM, gestational hypertension (GH), cardiovascular disease, thyroid disorders, coagulation abnormalities, etc.), lifestyle factors (smoking, alcohol consumption, etc.), and obstetric history (gravidity, parity, etc.). The questionnaire was independently reviewed and validated by two designated follow-up staff members at our center to ensure accuracy and completeness.

#### 2.2.2. Ultrasonographic Hemodynamic Parameters

Ultrasound and Doppler measurements were performed using the Voluson E8/E10 system (GE Healthcare Ultrasound, Milwaukee, WI, USA) with a probe frequency of 2–8 MHz. All ultrasonographic hemodynamic parameters were collected by two experienced physicians (X.H.Z, X.M.Z) in line with the ISUOG Practice Guidelines [15]. All ultrasound and Doppler examinations were performed before delivery or pregnancy termination. Thus, the ultrasound assessors were unaware of the subsequent pregnancy outcomes at the time of image acquisition and measurement, although they were not blinded to the diagnosis of UAT or to the contemporaneously available clinical information. However, because of the retrospective design, interobserver and intraobserver reproducibility were not formally evaluated. Gestational age (GA) was determined based on the last menstrual period and confirmed by crown–rump length measurements obtained during the nuchal translucency scan at 11–14 weeks.

Peak systolic velocity (PSV) and end-diastolic velocity of the UA, MCA and uterine artery were estimated using system-integrated software. Commonly used Doppler indices, including the pulsatility index (PI) and resistance index (RI) for the UA and MCA, were calculated. The cerebroplacental ratio (CPR) was derived as the PI of the MCA divided by the PI of the UA. For DV assessment, the pulsatility index for veins (PIV) was calculated using the formula PIV = (Vs − Va)/the time-averaged maximum velocity, where Vs represents the peak forward velocity during ventricular systole, and Va denotes the lowest-forward or peak-reversed velocity during atrial contraction (the a-wave).

Gestational age-specific reference values were obtained from parameter-specific published reference studies: the reference values for UA-PI, MCA-PI and CPR were from Gómez et al. [16], and those for mean UtA-PI from Drukker et al. [17]. MCA-PSV was expressed as multiples of the median [18].

### 2.3. Diagnostic Criteria

Diagnostic clues for UAT included [19]: (1) detection of two UA in the first or early second trimester by color Doppler imaging; (2) in the second and third trimesters, identification of a single UA and one vein on cross-sectional imaging of the UC and absence of paravesical UA flow on color Doppler, accompanied by hyperechoic thromboembolic material within a segment of the UA.

The FGR definition adopted the criteria developed by Gordijn et al. [20]: (1) the estimated fetal weight or abdominal circumference less than the 3rd percentile for GA; and (2) meeting two of the following four criteria—(1) the estimated fetal weight or abdominal circumference < the 10th percentile; (2) absent or reversed end-diastolic flow in the UA; (3) CPR < the 5th percentile; or (4) Uterine Artery PI > the 95th percentile.

Placental abnormalities included placentomegaly, marginal cord insertion, membranous placenta, and premature placental aging. We defined adverse pregnancy outcomes as intrauterine death and NICU admission [4].

### 2.4. Inclusion and Exclusion Criteria

Inclusion criteria included: (1) availability of at least one set of fetal biometry and Doppler measurements prior to delivery; (2) absence of structural and chromosomal abnormalities during the perinatal period; and (3) complete perinatal and neonatal follow-up data.

Exclusion criteria included: (1) major structural fetal anomalies or confirmed genetic abnormalities; (2) incomplete fetal hemodynamic data; and (3) missing follow-up data on fetal or neonatal outcomes.

### 2.5. Clinical Management

Post-diagnostic management, including the selection of urgent delivery or expectant management, was guided by gestational age, fetal hemodynamic status, and electronic fetal heart-rate monitoring, according to our previously reported protocol [4]. Briefly, expectantly managed pregnancies underwent electronic fetal monitoring every 2 days, weekly Doppler assessment, and close fetal-movement surveillance, with supportive treatment and antenatal corticosteroids administered when clinically indicated.

### 2.6. Statistics

All statistical analyses were performed using R software (version 4.4.1; R Foundation for Statistical Computing, Vienna, Austria). Statistical significance was considered as *p* < 0.05 for two-sided tests. The Shapiro–Wilk test was used to assess the distribution of continuous variables. Normally distributed quantitative data were presented as mean ± standard deviation (SD) and compared between groups using the independent samples *t*-test. Non-normally distributed continuous variables were expressed as median (interquartile range, IQR) and compared using the Mann–Whitney U test. Categorical variables were summarized as numbers and percentages [*n* (%)] and compared using Pearson’s χ^2^ test or Fisher’s exact test.

The aim of this study was to develop a prediction model for adverse pregnancy outcomes among pregnancies complicated by UAT. Candidate predictors were selected a priori based on previous literature, clinical knowledge, and their potential associations with adverse pregnancy outcomes in UAT pregnancies, rather than solely based on univariable statistical significance. The candidate variables included maternal characteristics (maternal age, pre-pregnancy body mass index, gravidity, and parity), UAT-related factors (gestational age at UAT diagnosis), fetal and placental hemodynamic parameters assessed by Doppler ultrasonography (UA-PI, MCA-PSV, MCA-PI, DV-PIV, and UtA-PI), and clinical factors potentially associated with perinatal outcomes (diabetes, hypertension, thyroid disorders, FGR, and laterality of UAT).

The 15 prespecified candidate predictors were subsequently entered into a least absolute shrinkage and selection operator (LASSO) regression model for variable selection. Ten-fold cross-validation was applied to determine the optimal penalty parameter (λ), thereby reducing the risk of overfitting and identifying predictors with the greatest predictive value. Variables selected by LASSO regression were then incorporated into a multivariable logistic regression model to establish the final prediction model for adverse pregnancy outcomes. The logistic regression model is expressed as follows:

logit(*P*) = β_0_ + β_1_X_1_ + β_2_X_2_ + … + β_n_X_n_

where *P* represents the predicted probability of adverse pregnancy outcomes, β_0_ represents the intercept, and β_n_ represents the regression coefficient of each predictor. A nomogram was subsequently developed based on the final logistic regression model to provide individualized risk prediction.

Model performance was evaluated in terms of discrimination, calibration, and clinical utility. Discrimination was assessed using the receiver operating characteristic (ROC) curve and the area under the curve (AUC). The optimal prediction threshold was determined according to the maximum Youden index, and the corresponding sensitivity and specificity were calculated.

Model calibration was assessed using the calibration curve, calibration intercept, calibration slope, and Brier score. The calibration intercept and slope were used to evaluate the agreement between predicted probabilities and observed outcomes. Internal validation was performed using 1000 bootstrap resamples. Optimism-adjusted performance measures, including the optimism-adjusted C-index, calibration intercept, and calibration slope, were calculated to evaluate model stability and correct for potential optimism caused by model overfitting.

Decision curve analysis (DCA) was performed to assess the potential clinical utility of the prediction model. The net benefit across different threshold probabilities was calculated and compared between the nomogram model, the “treat-all” strategy, and the “treat-none” strategy.

## 3. Results

### 3.1. General Characteristics

**Figure 1:**
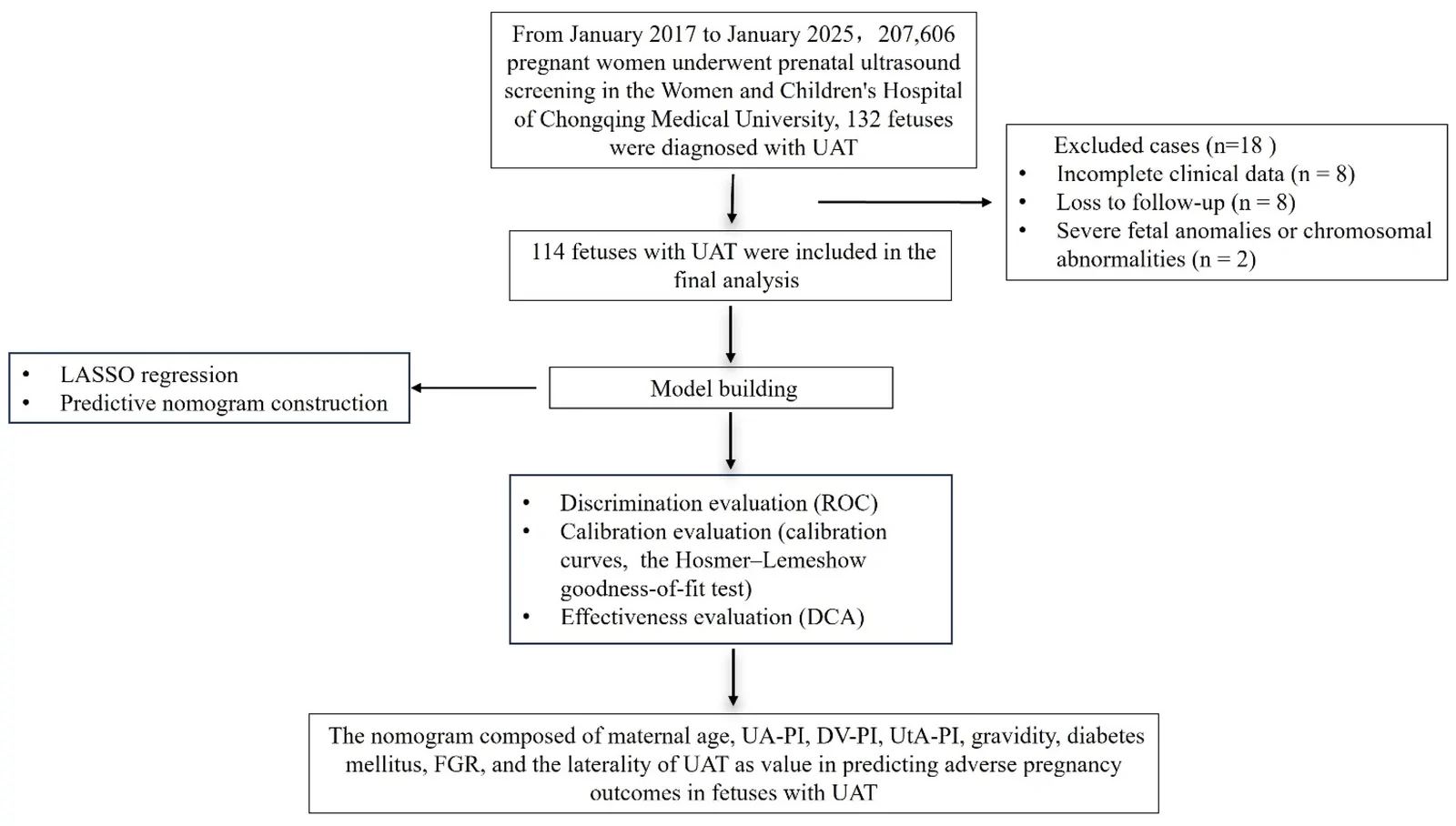
Flow chart for patient selection. GA, gestational age; FGR, fetal growth restriction; UA-PI, the pulsatility index (PI) for umbilical artery (UA); ROC, receiver operative characteristic; DCA, decision curve analysis.

A total of 207,606 pregnant women underwent prenatal ultrasound screening at our center from January 2017 to January 2025, among whom 132 fetuses were diagnosed with UAT. Eighteen cases were excluded due to incomplete clinical data (*n* = 8), loss to follow-up (*n* = 8), or severe fetal anomalies or chromosomal abnormalities (*n* = 2). Ultimately, 114 fetuses with UAT were included in the final analysis (Figure 1), including 57 patients with adverse pregnancy outcomes and 57 patients without adverse pregnancy outcomes. The baseline characteristics of the study population are presented in Table 1.

Compared with the non-adverse pregnancy group, patients with adverse pregnancy outcomes had significantly lower gestational age at delivery (median [IQR]: 34.10 [29.40, 35.50] vs. 38.10 [36.50, 39.00] weeks, *p* < 0.001) and shorter duration of pregnancy prolongation (median [IQR]: 2.00 [1.00, 14.00] vs. 17.00 [5.00, 74.00] days, *p* = 0.001). In addition, Apgar scores at 1 and 5 min were significantly lower in the adverse pregnancy outcome group (*p* < 0.001 and *p* = 0.001, respectively).

Regarding Doppler ultrasound parameters, the adverse pregnancy outcome group showed higher DV-PIV (median [IQR]: 0.67 [0.54, 1.07] vs. 0.56 [0.48, 0.70], *p* = 0.007) and higher UtA-PI (median [IQR]: 0.64 [0.56, 0.72] vs. 0.56 [0.50, 0.65], *p* = 0.010). Although not statistically significant, CPR tended to be lower in patients with adverse pregnancy outcomes (1.80 [0.72] vs. 2.01 [0.52], *p* = 0.076).

For clinical characteristics, umbilical cord abnormalities were more frequent among patients with adverse pregnancy outcomes (59.6% vs. 26.3%, *p* = 0.001), and FGR was also more common in this group (26.3% vs. 5.3%, *p* = 0.005). The distribution of UAT laterality differed significantly between groups (*p* = 0.019), with a higher proportion of right-sided thrombosis observed in the adverse pregnancy outcome group (47.4% vs. 24.6%). No significant differences were observed between groups regarding maternal age, pre-pregnancy BMI, gestational weight gain, parity, gravidity, uterine artery PI, UA-PI, MCA Doppler parameters, GDM, GH, thyroid disorders, coagulation abnormalities, or placental abnormalities (all *p* > 0.05).

### 3.2. Performance Evaluation of Predictive Models

Based on the 15 prespecified candidate predictors, LASSO regression was performed for variable selection, with ten-fold cross-validation used to determine the optimal penalty parameter. As shown in Supplementary Figure S1, the model achieved the minimum binomial deviance at λ_min_, with eight variables retaining non-zero coefficients. Under the more stringent λ_1se_ criterion, four variables were retained. Considering both predictive performance and clinical interpretability, the eight predictors identified under the λ_min_ criterion were further included in the subsequent multivariable logistic regression analysis. The coefficient profiles of candidate predictors under different penalty parameters are presented in Supplementary Figure S2.

The variables selected by LASSO regression and supported by clinical relevance were further incorporated into the multivariable logistic regression model. As shown in Table 2, UA-PI, DV-PI, FGR, and UAT laterality were associated with the risk of adverse pregnancy outcomes. Specifically, increased UA-PI was significantly associated with a higher risk of adverse pregnancy outcomes (OR = 7.15, 95% CI: 1.01–50.57, *p* = 0.049). Similarly, elevated DV-PI was significantly associated with adverse pregnancy outcomes (OR = 13.18, 95% CI: 2.34–74.41, *p* = 0.003). Compared with patients without FGR, patients with FGR had a significantly increased risk of adverse pregnancy outcomes (OR = 7.53, 95% CI: 1.77–31.97, *p* = 0.006). In addition, UAT laterality showed a borderline association with adverse pregnancy outcomes (OR = 2.61, 95% CI: 1.00–6.81, *p* = 0.050). Other predictors included in the model, including maternal age, UtA-PI, gravidity, and diabetes, did not reach statistical significance after multivariable adjustment.

**Figure 2:**
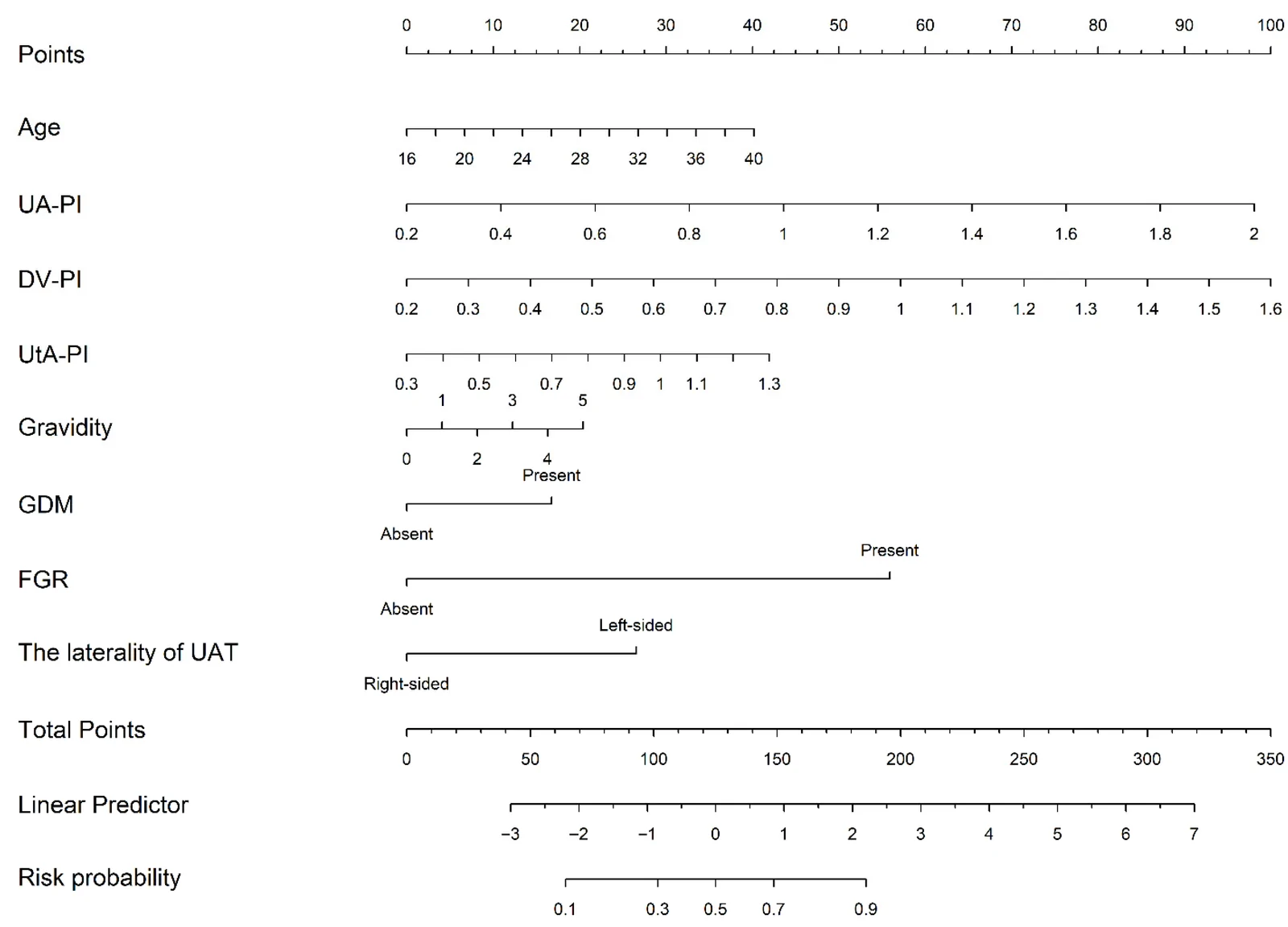
Nomogram for the prediction of adverse outcomes in fetuses with UAT. GA, gestational age; UA-PI, the pulsatility index (PI) for umbilical artery (UA); FGR, fetal growth restriction.

Based on the final multivariable logistic regression model, a nomogram was developed to predict the probability of adverse pregnancy outcomes among patients with UAT (Figure 2). The model incorporated eight predictors, including maternal age, UA-PI, DV-PI, UtA-PI, gravidity, diabetes, FGR, and UAT laterality. By converting each predictor into corresponding points and summing the total points, the individualized predicted probability of adverse pregnancy outcomes could be estimated.

**Figure 3:**
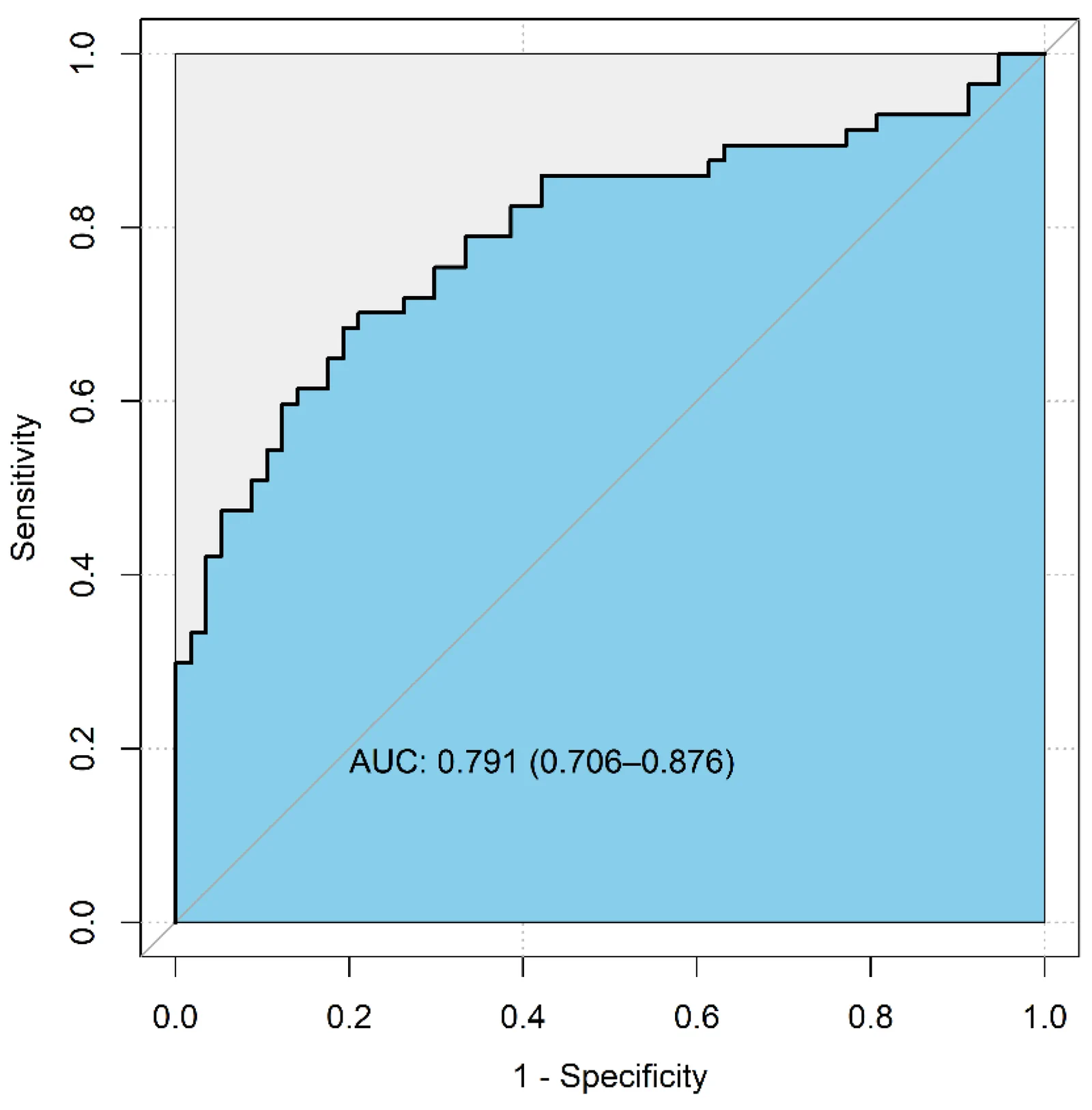
Receiver operative characteristic curves of the nomogram. AUC, area under the ROC curve.

The discriminative performance of the nomogram was evaluated using receiver operating characteristic (ROC) curve analysis (Figure 3). The model showed good discrimination, with an area under the curve (AUC) of 0.791 (95% CI: 0.706–0.876). The optimal cutoff value was 0.509, corresponding to a sensitivity of 70.2%, specificity of 78.9%, and Youden index of 0.491 (Supplementary Table S1).

**Figure 4:**
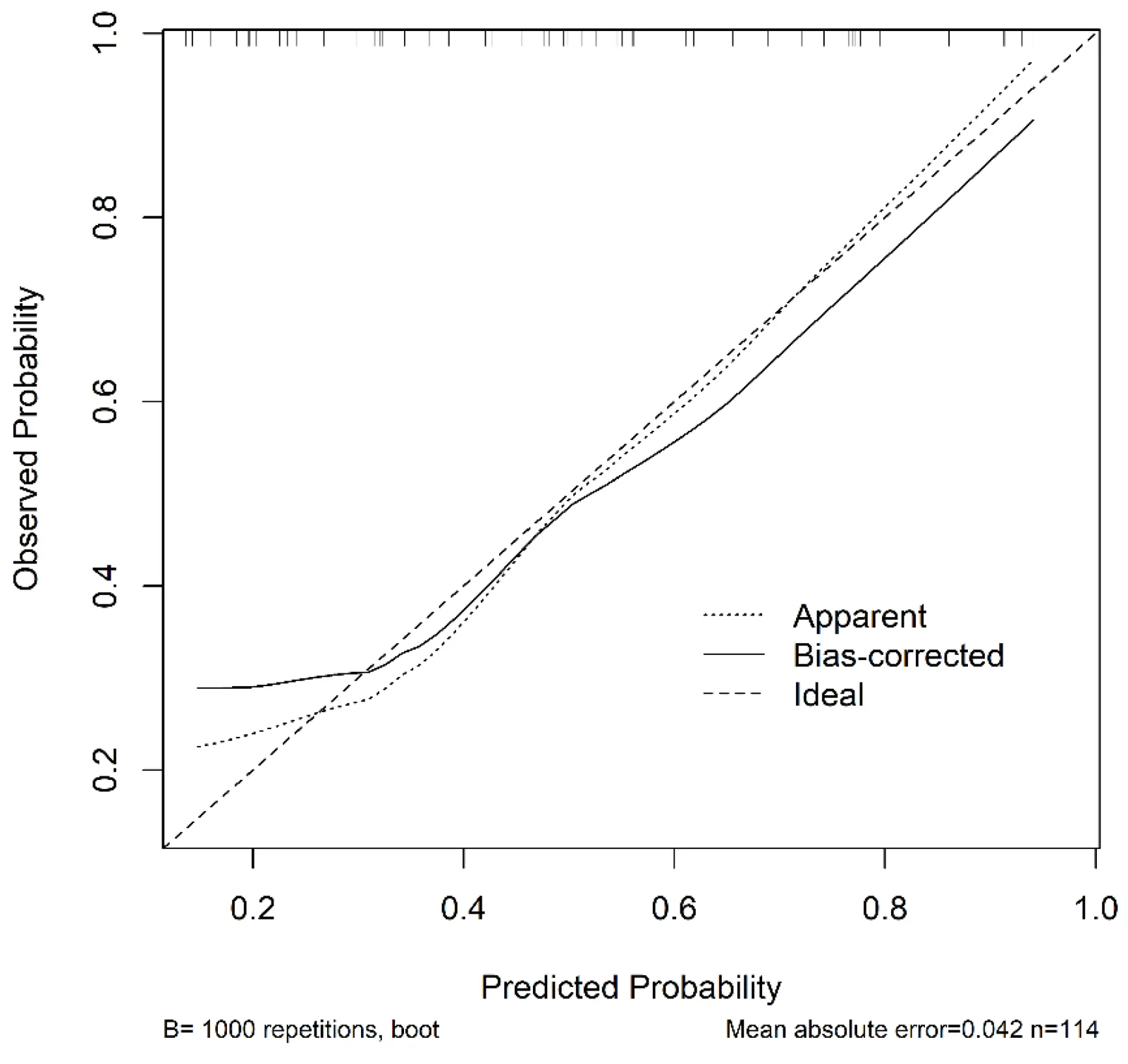
Calibration curve for predicting adverse outcomes in fetuses with UAT.

Calibration performance was assessed using 1000 bootstrap resamples (Figure 4). The calibration curve demonstrated good agreement between predicted and observed probabilities, with a mean absolute error of 0.042, indicating satisfactory calibration of the model.

**Figure 5:**
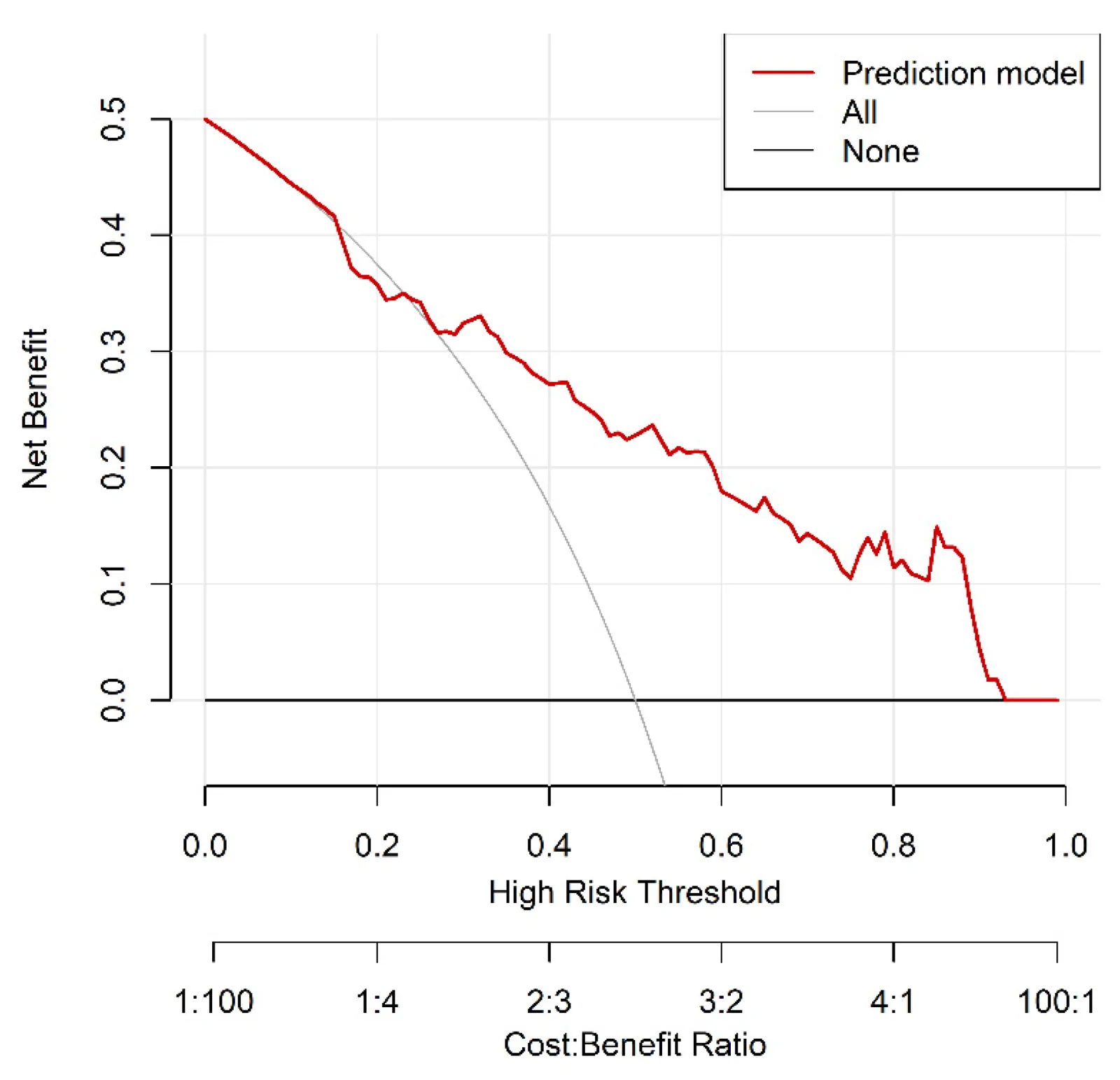
Decision curve analysis in predicting adverse outcomes in fetuses with UAT.

Decision curve analysis demonstrated that the nomogram provided greater net benefit than both the “treat-all” and “treat-none” strategies across a threshold probability range of 11–92% (Figure 5), suggesting its potential clinical utility for identifying UAT pregnancies at increased risk of adverse pregnancy outcomes. The performance of the prediction model was further evaluated in terms of discrimination, calibration, and internal validation (Supplementary Table S2). The apparent C-index of the model in the development cohort was 0.791. The apparent calibration intercept was 0.00, the calibration slope was 1.00, and the Brier score was 0.183. After internal validation using 1000 bootstrap resamples, the optimism-adjusted C-index decreased to 0.737, with an optimism-adjusted calibration intercept of −0.041, calibration slope of 0.711, and Brier score of 0.215. These findings indicate that the model showed some degree of optimism due to potential overfitting; however, it maintained stable predictive performance after optimism correction.

## 4. Discussion

As thrombosis acutely compromises fetoplacental circulation, it may trigger a cascade of ischemic events similar to those seen in maternal vascular malperfusion syndromes [19,21,22]. Based on this, we developed and validated a comprehensive model that integrates maternal clinical features, comorbid conditions, fetal baseline characteristics, and Doppler-derived hemodynamic parameters. Notably, the inclusion of hemodynamic indices—reflecting real-time fetal physiological status—significantly improved the predictive performance. These findings highlight the feasibility and clinical value of a multi-dimensional predictive framework for early risk stratification and personalized clinical decision-making.

The pathogenesis of UAT may be associated with endothelial damage, particularly disorders of glucose metabolism and coagulation dysfunction [3,23,24]. In our model, we incorporated maternal comorbidities such as GH, GDM, thyroid disorders, and coagulopathies. Additionally, we included variables potentially affecting maternal and placental vascular remodeling, as well as fetal nutritional status and intrauterine environment, including gestational weight gain, placental abnormalities, and nuchal cord entanglement. Nevertheless, none of these clinical variables demonstrated statistically significant differences between the adverse and non-adverse outcome groups in either the training or validation cohorts. Although factors such as maternal age, GH, GDM, coagulation disorders, and gestational weight gain were retained through LASSO regression, none exhibited independent predictive value after adjustment for potential confounders.

Notably, none of the fetuses with UAT complicated by FGR exhibited absent or reversed end-diastolic flow (AREDF) in the UA in our previous cohort [4], suggesting that FGR may result from insufficient compensatory perfusion via the remaining patent UA, rather than overt hemodynamic compromise. Based on this, the present study incorporated the presence of FGR as a predictive variable—rather than defining it as an adverse outcome—marking a methodological distinction from previous models. Patients with FGR had a significantly increased risk of adverse pregnancy outcomes compared with those without FGR (OR = 7.53, 95% CI: 1.77–31.97, *p* = 0.006). These findings highlight that the coexistence of FGR in UAT fetuses substantially elevates the risk of adverse outcomes and warrants intensified monitoring and timely clinical intervention.

Given that it is characterized by the presence of only one functioning umbilical artery, UAT is considered a specific variant of SUA. The most common hemodynamic alteration in fetuses with UAT involved the UA [4], which typically showed elevated diastolic flow, rather than AREDF. Contro et al. [25] proposed that reduced UA-PI in fetuses with a single umbilical artery may represent an early adaptive response involving compensatory dilatation of the solitary artery to maintain fetal perfusion. In the present study, UA-PI was analyzed as a continuous variable, and each 1-unit increase in UA-PI was associated with a 7.15-fold increase in the odds of adverse pregnancy outcomes (95% CI, 1.01–50.57). From this perspective, UA-PI was modeled continuously in the present study because values at the lower and upper extremes may have different physiological implications in UAT, with an elevated UA-PI in UAT possibly indicating inadequate or exhausted vascular compensation.

The DV-PIV has been recognized as a key marker of right ventricular diastolic function [11,26,27]. Consistent with this, prior research at our center found that all seven cases of UAT-related intrauterine fetal demise exhibited abnormal a-waves in the DV. In the present study, DV-PIV was significantly higher in fetuses with adverse pregnancy outcomes than in those without adverse outcomes (median [IQR], 0.67 [0.54–1.07] vs. 0.56 [0.48–0.70]; *p* = 0.007). DV-PIV was retained by LASSO and remained independently associated with adverse pregnancy outcomes in the multivariable logistic regression model (OR, 13.18; 95% CI, 2.34–74.41; *p* = 0.003). These findings suggest that increased impedance to venous return and impaired right ventricular diastolic function may reflect progressive fetal hemodynamic compromise in UAT.

Several limitations should be acknowledged in this study. First, due to the sudden and unpredictable nature of UAT, GA at diagnosis may not accurately reflect the true onset of the condition. Additionally, hemodynamic parameters provide only a single time-point assessment and may not fully capture the dynamic nature of fetal circulation. Second, because of the rarity of UAT, external validation using data from other prenatal diagnostic centers was not conducted. Although the model demonstrated strong predictive performance, the possibility of false-positive predictions cannot be excluded. Therefore, in cases complicated by FGR and abnormal UA-PI, timely intervention should be guided by a comprehensive assessment, which may help prevent avoidable fetal loss in the late gestational period.

Given the rarity and unpredictable clinical course of UAT, management decisions are often based primarily on individual clinical experience. The proposed clinical prediction model may provide a more objective approach to early risk stratification, particularly with the inclusion of UA-PI, and facilitate individualized surveillance and delivery planning. External validation in multicenter cohorts is warranted before routine clinical implementation.

## Figures and Tables

**Table 1:** Baseline characteristics of all patients.

Variable		Non-Adverse Pregnancy (*n* = 57)	Adverse Pregnancy (*n* = 57)	*p*-Value
Maternal age		29.53 (3.91)	30.44 (5.21)	0.293
Gravidity		1.00 [0.00, 1.00]	1.00 [0.00, 2.00]	0.771
Parity		0.00 [0.00, 0.00]	0.00 [0.00, 0.00]	0.791
GA at diagnosis		31.10 [28.00, 37.00]	31.60 [25.50, 34.10]	0.191
GA at delivery		38.10 [36.50, 39.00]	34.10 [29.40, 35.50]	<0.001
Height		159.63 (4.70)	158.53 (5.06)	0.23
Pre-pregnancy weight		55.20 (7.36)	53.38 (7.96)	0.207
Duration of pregnancy prolongation		17.00 [5.00, 74.00]	2.00 [1.00, 14.00]	0.001
Pre-pregnancy BMI		21.29 [19.88, 23.14]	20.62 [19.23, 23.51]	0.418
BMI at delivery		26.35 [24.03, 28.84]	25.32 [22.74, 27.22]	0.1
Gestational weight gain		12.11 (4.48)	10.56 (4.83)	0.078
UA-PI		0.67 [0.57, 0.82]	0.73 [0.59, 0.87]	0.106
MCA-PSV		55.81 [48.87, 62.63]	55.33 [47.76, 62.53]	0.391
MCA-PI		1.37 [1.12, 1.53]	1.24 [0.96, 1.51]	0.256
CPR		2.01 (0.52)	1.80 (0.72)	0.076
DV-PIV		0.56 [0.48, 0.70]	0.67 [0.54, 1.07]	0.007
UtA-PI		0.56 [0.50, 0.65]	0.64 [0.56, 0.72]	0.01
Length of UC		50.00 [50.00, 60.00]	50.00 [45.00, 60.00]	0.073
UC abnormality				0.001
	Absent	42 (73.7)	23 (40.4)	
	Present	15 (26.3)	34 (59.6)	
Nuchal cord				0.024
	None	35 (61.4)	48 (84.2)	
	1	17 (29.8)	7 (12.3)	
	≥2	5 (8.8)	2 (3.5)	
GDM				0.514
	Absent	45 (78.9)	41 (71.9)	
	Present	12 (21.1)	16 (28.1)	
GH				1
	Absent	52 (91.2)	52 (91.2)	
	Present	5 (8.8)	5 (8.8)	
Alcohol consumption				/
	Absent	57 (100.0)	57 (100.0)	
	Present	0 (0.0)	0 (0.0)	
Smoking				/
	Absent	57 (100.0)	57 (100.0)	
	Present	0 (0.0)	0 (0.0)	
Thyroid disorders				1
	Absent	50 (87.7)	51 (89.5)	
	Present	7 (12.3)	6 (10.5)	
Coagulation abnormalities				0.242
	Absent	54 (94.7)	57 (100.0)	
	Present	3 (5.3)	0 (0.0)	
FGR				0.005
	Absent	54 (94.7)	42 (73.7)	
	Present	3 (5.3)	15 (26.3)	
Placental abnormality				0.127
	Absent	57 (100.0)	53 (93.0)	
	Present	0 (0.0)	4 (7.0)	
Laterality of UAT				0.019
	Left	43 (75.4)	30 (52.6)	
	right	14 (24.6)	27 (47.4)	
Apgar—1 min		10.00 [10.00, 10.00]	10.00 [9.00, 10.00]	<0.001
Apgar—5 min		10.00 [10.00, 10.00]	10.00 [10.00, 10.00]	0.001

BMI = body mass index; GA = gestational age; UC = umbilical cord; UAT = umbilical artery thrombosis; FGR = fetal growth restriction; UA = umbilical artery; PI = pulsatility index; MCA = middle cerebral artery; PSV = peak systolic velocity; CPR = cerebroplacental ratio; DV = ductus venosus; PIV = pulsatility index for veins; UtA = uterine artery; GDM = gestational diabetes mellitus; GH = gestational hypertension.

**Table 2:** Multivariate logistic regression analyses for predictors.

Variables	β	SE	OR	OR (95% CI)	*p*-Value
Age	0.06	0.06	1.06	1.06 (0.95, 1.19)	0.287
Gravidity	0.15	0.19	1.16	1.16 (0.80, 1.68)	0.441
Diabetes	0.6	0.53	1.83	1.83 (0.65, 5.16)	0.252
FGR	2.02	0.74	7.53	7.53 (1.77, 31.97)	0.006
Laterality of UAT	0.96	0.49	2.61	2.61 (1.00, 6.81)	0.05
UA-PI	1.97	1	7.15	7.15 (1.01, 50.57)	0.049
DV-PIV	2.58	0.88	13.18	13.18 (2.34, 74.41)	0.003
UtA-PI	1.51	1.62	4.55	4.55 (0.19, 109.68)	0.351

UA = umbilical artery; PI = pulsatility index; DV = ductus venosus; PIV = pulsatility index for veins; UtA = uterine artery; FGR = fetal growth restriction; UAT = umbilical artery thrombosis.

## Data Availability

The data that support the findings of our study are included in the article and Supplementary Materials. The raw data in this study are not publicly available in order to protect participant confidentiality, but are available from the corresponding author upon reasonable request. If you wish to request access to the data, please contact Prof. Suzhen Ran (ransuzhen0000@163.com) at the Department of Ultrasound, Chongqing Health Center for Women and Children, Chongqing, China.

## Supplementary Materials

The following supporting information can be downloaded at: https://www.mdpi.com/article/10.3390/jcm15176779/s1, Supplementary Figure S1: Ten-fold cross-validation for selecting the optimal penalty parameter (λ) in the LASSO regression model; Supplementary Figure S2: LASSO coefficient profiles of the candidate predictors. Supplementary Table S1: Multivariate logistic regression analyses for predictors. Supplementary Table S2: Performance and internal validation of the prediction model.
