# Supplementary material for: Development and Validation of a Prenatal Doppler Hemodynamic–Clinical Risk Model for Predicting Adverse Perinatal Outcomes in Fetuses with Umbilical Artery Thrombosis
Source: J Clin Med. 2026 Sep 1;15(17):6779. doi: 10.3390/jcm15176779 (PMC13566282; doi:10.3390/jcm15176779)
Supplement: Supplementary file 1 [file jcm-15-06779-s001.zip › Table S1.pdf]

**Supplementary Table S1. Multivariate logistic regression analyses for predictors**

| Variables | β | SE | OR | Lower 95%CI | Upper 95%CI | OR (95% CI) | *P* value |
|---|---|---|---|---|---|---|---|
| Intercept | -6.85 | 2.28 | 0 | 0 | 0.09 | 0.00 (0.00, 0.09) | 0.003 |
| Age | 0.06 | 0.06 | 1.06 | 0.95 | 1.19 | 1.06 (0.95, 1.19) | 0.287 |
| Gravidity | 0.15 | 0.19 | 1.16 | 1.01 | 50.57 | 1.16 (0.80, 1.68) | 0.441 |
| Diabetes | 0.6 | 0.53 | 1.83 | 2.34 | 74.41 | 1.83 (0.65, 5.16) | 0.252 |
| FGR | 2.02 | 0.74 | 7.53 | 0.19 | 109.68 | 7.53 (1.77, 31.97) | 0.006 |
| The laterality of UAT | 0.96 | 0.49 | 2.61 | 0.8 | 1.68 | 2.61 (1.00, 6.81) | 0.05 |
| UA-PI | 1.97 | 1 | 7.15 | 0.65 | 5.16 | 7.15 (1.01, 50.57) | 0.049 |
| DV-PIV | 2.58 | 0.88 | 13.18 | 1.77 | 31.97 | 13.18 (2.34, 74.41) | 0.003 |
| UtA-PI | 1.51 | 1.62 | 4.55 | 1 | 6.81 | 4.55 (0.19, 109.68) | 0.351 |

UA= umbilical artery; PI= pulsatility index; DV= ductus venosus; PIV= pulsatility index for veins;
UtA= uterine artery; FGR= fetal growth restriction;UAT= umbilical artery thrombosis

| threshold | sensitivity | specificity | Youden |
|---|---|---|---|
| 0.50852204 | 0.70175439 | 0.78947368 | 0.49122807 |

The general form of the logistic regression model was:

$logit(P)=\beta_0+\beta_1X_1+\beta_2X_2+....+\beta_nX_n$

The final prediction model was developed using multivariable logistic regression. The linear predictor for the probability of an adverse pregnancy outcome was calculated as follows:

logit(P)=-6.85+0.06(Age)+1.97(UA-PI)+2.58(DV-PI)+1.51(UtA-PI)+0.15(Gravidity)+0.60(Diabetes)+2.02(FGR)+0.96(Laterality of UAT)

where *P* represents the predicted probability of an adverse pregnancy outcome. The predicted probability was obtained using the following transformation:

$$P=\frac{1}{1+e^{-logit(P)}}$$
