# Supplementary material for: Development and Validation of a Prenatal Doppler Hemodynamic–Clinical Risk Model for Predicting Adverse Perinatal Outcomes in Fetuses with Umbilical Artery Thrombosis
Source: J Clin Med. 2026 Sep 1;15(17):6779. doi: 10.3390/jcm15176779 (PMC13566282; doi:10.3390/jcm15176779)
Supplement: Supplementary file 1 [file jcm-15-06779-s001.zip › Table S2.pdf]

Supplementary Table S2. Performance and internal validation of the prediction model

Performance measure	Apparent estimate	Optimism-adjusted estimate
AUC/C-index	0.791	0.737
Calibration intercept	0.000	-0.040
Calibration slope	1.000	0.711
Brier score	0.183	0.215
